# Effects of foam roller on pain intensity in individuals with chronic and acute musculoskeletal pain: a systematic review of randomized trials

**DOI:** 10.1186/s12891-024-07276-6

**Published:** 2024-02-24

**Authors:** Inaê Silva Santos, Almir Vieira Dibai-Filho, Patrícia Gabrielle dos Santos, José Djalma Arrais Júnior, Daniella Dias de Oliveira, Daniel Santos Rocha, Cid André Fidelis-de-Paula-Gomes

**Affiliations:** 1 Postgraduate Program in Rehabilitation Sciences, Nove de Julho University, São Paulo, SP Brazil; 2 Postgraduate Program in Physical Education, Universidade Federal do Maranhão, São Luís, MA Brazil; 3 Programa de Pós-Graduação em Ciências da Reabilitação, Universidade Nove de Julho, Rua Vergueiro, 235/249, Liberdade, São Paulo, SP CEP 01504- 001 Brazil

**Keywords:** Pain measurement, Pain, Musculoskeletal pain, Pain intensity

## Abstract

**Objective:**

To analyze the effects of using foam roller on pain intensity in individuals with chronic and acute musculoskeletal pain.

**Methods:**

This systematic review was registered in the National Institute for Health Research’s prospective online registry of systematic reviews (PROSPERO) under CRD42023456841. The databases Pubmed, Medline (via Ovid), Embase, BVS, and PEDro (Physiotherapy Evidence Database) were consulted to carry out this systematic review. Notably, the records of clinical trials characterized as eligible were manually searched. The search terms were: (foam rolling OR foam rolling vibration) AND (acute musculoskeletal pain) AND (chronic musculoskeletal pain). The search was performed until August 22, 2023. For the analysis of the methodological quality, the PEDro scale was used for each of the manuscripts included in the systematic review. Due to the heterogeneity in the studies included in this systematic review, performing a meta-analysis of the analyzed variables was impossible.

**Results:**

Only six manuscripts were eligible for data analysis. The type of FR used was non-vibrational, being applied by a therapist in only one of the manuscripts. With an application time ranging from at least 45 s to 15 min, the non-vibrational FR was applied within a day up to six weeks. Using the PEDro scale, scores were assigned that varied between 4 and 8 points, with an average of 6 ± 1.29 points. Only two randomized clinical trials found a significant benefit in pain intensity of adding FR associated with a therapeutic exercise protocol in individuals with patellofemoral pain syndrome and chronic neck pain.

**Conclusion:**

The results of this systematic review do not elucidate or reinforce the clinical use of FR in pain intensity in individuals with chronic and acute musculoskeletal pain.

## Introduction

Foam roller (FR) is a popular self-massage or self-myofascial release technique. It involves using a foam roller, a tube-shaped device composed of or surrounded by foam, to apply pressure on the affected body parts [1]. FR has been found to have various benefits for athletes and individuals engaged in physical activity. One of the main benefits of FR is its ability to reduce muscle soreness and improve recovery. FR reduced muscle soreness and increased voluntary muscle activation, vertical jump height, and flexibility compared to a control group [2]. Similar to these findings, the meta-analysis by Wiewelhove et al. [3] concluded that FR could alleviate muscle fatigue and soreness, making it an effective intervention for post-exercise recovery.

FR has also improved range of motion (ROM) and flexibility. Promises significant increases in knee-joint ROM after just two 1-minute [4]. Another study by Shu et al. [5] highlighted the physiological benefits of FR, including improved ROM, reduced risk of sports injury, and shortened recovery period [5]. In addition to its effects on muscle soreness and flexibility, FR has been found to have neurophysiological effects. Young et al. [1] investigated the effects of roller massage, which includes foam rolling, on spinal excitability and found that it decreased spinal excitability in the soleus muscle [1]. This suggests that FR may have a positive impact on the neuromuscular system.

Despite the reported clinical effects, there is a clear need for further research to fully elucidate the specific physiological mechanisms underlying the effects of FR. Despite this context, some physiological effects have been reported over the last few years. Among the most prominent is the constant tension on soft tissues, which overloads the skin receptors, modulating pain, and stretching. Increased local blood flow promotes the modulation of inflammation in the fascia. Increase in circulating neutrophils and the activity of alpha motor neurons, and a decrease in neural inhibition, facilitating the communication of afferent receptors in the connective tissue [6].

Specifically, FR has improved blood circulation and arterial function. Pablos et al. [7] observed increased blood flow and muscle oxygen saturation after foam rolling, contributing to tissue healing and muscle recovery [7]. Additionally, FR has been shown to decrease arterial stiffness and increase nitric oxide concentration, further supporting its positive effects on arterial function [7].

Overall, FR is a beneficial technique for athletes and individuals engaged in physical activity. It can help reduce muscle soreness, improve recovery, increase range of motion and flexibility, and positively affect the neuromuscular system and arterial function. Incorporating FR into a regular exercise routine may enhance performance and prevent injury. Among the various physiological mechanisms proposed to justify the use of FR, attention is drawn to the one that highlights the modulation of pain in the central nervous system using FR. The pressure exerted by the FR on the soft tissues would promote an overload on the skin receptors, causing the inhibition of pain sensation and tolerance to stretch [8–11].

However, although a clear physiological relationship exists, the potential effects and clinical benefits of using FR for pain in acute and chronic musculoskeletal conditions have yet to be fully elucidated. Mainly because, to date, previously published systematic reviews, with or without meta-analysis, on the use of FR have included the analysis of healthy participants. Therefore, they did not explore FR’s effects and clinical repercussions on pain intensity in individuals with acute or chronic musculoskeletal conditions. These gaps, added to the growing clinical use of FR, justify the preparation and carrying out of this systematic review.

Therefore, this study aims to systematically review the literature on the effects of using FR on pain intensity in individuals with chronic and acute musculoskeletal pain. Thus, this review hypothesizes that using FR associated with exercise protocols improves pain intensity in individuals with chronic musculoskeletal conditions.

## Methodology

This systematic review was carried out based on the guidelines provided by PRISMA (Preferred Reporting Items for Systematic Reviews and Meta-Analysis) [12]. It was registered in the National Institute for Health Research’s prospective online registry of systematic reviews (PROSPERO: https://crd.york.ac.uk/PROSPERO/) under CRD42023456841.

Systematic searches were performed in the following databases: Pubmed, Medline (via Ovid), Embase, BVS, and PEDro. Notably, the records of clinical trials characterized as eligible were manually searched.

The search terms were: (foam rolling OR foam rolling vibration) AND (acute musculoskeletal pain) AND (chronic musculoskeletal pain). The search terms were defined by taking previously published systematic reviews as an example [3, 13, 14]. The date of the last survey was August 22, 2023.

## Eligibility criteria

Studies were considered for inclusion if they met the criteria:

- Randomized Clinical trials.
- Published in a peer-reviewed journal.
- English language.
- Individuals with chronic and acute musculoskeletal pain. The diagnosis of chronic pain was consistent with the British Pain Society definition (chronic pain that lasts beyond the time that tissue healing would usually be expected to have occurred, often taken as ≥ 3 months). The diagnosis of acute pain was consistent with the British Pain Society definition, often taken as ≤3 months) [15].
- Male and female, Aged > 18 years old.
- Intensity of Pain as a primary or secondary outcome. Pain intensity is measured with a Numeric Rating Scale (NRS) or a Visual Analogue Scale (VAS).
- For Interventions: foam rolling and/or foam rolling vibration and exercise therapy (exercise program involving warming-up, motor learning, balance coordination, strengthening, and stretching exercises).

Conference abstracts, in vivo and in vitro studies, systematic reviews and meta-analyses, case reports, experimental studies, and crossover studies, manuscripts composed of interventions other than foam rolling and/or foam rolling vibration and exercise therapy, and manuscripts that analyzed pain intensity related to delayed onset muscle soreness (DOMS) and/or did not assess pain intensity were excluded.

In addition, specific diagnoses such as radicular pain, radiculopathy, myelopathy, fracture, infection, dystonia, tumor, inflammatory disease, osteoporosis, fibromyalgia, and studies on mixed pain populations (e.g., spinal pain both from neck and back) were results for individuals with are not presented separately.

## Identification and selection of studies

The study screening process started with reading the titles and abstracts of the manuscripts. The potentially eligible manuscripts were read in total to complete the eligibility check.

In this way, a standardized data extraction form was used. The following data were extracted: Number of participants/gender, age, sample characteristics, evaluation time, pain assessment, therapy program, frequency of treatment, experimental group, control group, type of foam roller, characteristics of a foam roller, time, application mode, body region, combined therapy, characteristics, duration of treatment, control group post-intervention (mean ± standard deviation), experimental group post-intervention (mean ± standard deviation), results, conclusions.

Finally, an analysis of the methodological quality of the included studies was performed using the PEDro scale. Of 11 items, 10 are scored (items 2 to 11). The items are Random allocation, Concealed allocation, Baseline comparability, Blind subjects, Blind therapists, Blind assessors, Adequate follow-up, Intention-to-treat analysis, Between-group comparisons, Point estimates, and variability. The PEDro scale has good levels of validity and reliability, where higher scores mean higher methodological quality [16–18]. Scores for included manuscripts were taken directly from the PEDro database whenever possible. For the screening process, data extraction, and methodological quality analysis, when articles were not found or the score was not established, two independent (CAFDP and ISS) trained reviewers evaluated the article using the PEDro scale. In disagreement, a third reviewer (AVDF) was consulted to provide a consensus.

## Data analysis

Data on pain intensity and musculoskeletal pain variables were extracted from the studies selected for inclusion and structured according to their follow-up times. Time of follow-up was defined as immediately after treatment (≤ 1 day); short (up to 4 weeks), medium (up to 12 weeks), and long (> 12 weeks). To analyze the effect of interventions on pain intensity variables, the mean difference between the groups and the 95% confidence intervals for each study were extracted. When the study did not present the mean difference between the groups and the confidence intervals, both were calculated using the confidence interval calculator provided by PEDro.

Due to the heterogeneity in the studies included in this systematic review, performing a meta-analysis of the analyzed variables was impossible.

## Results

**Fig. 1:**
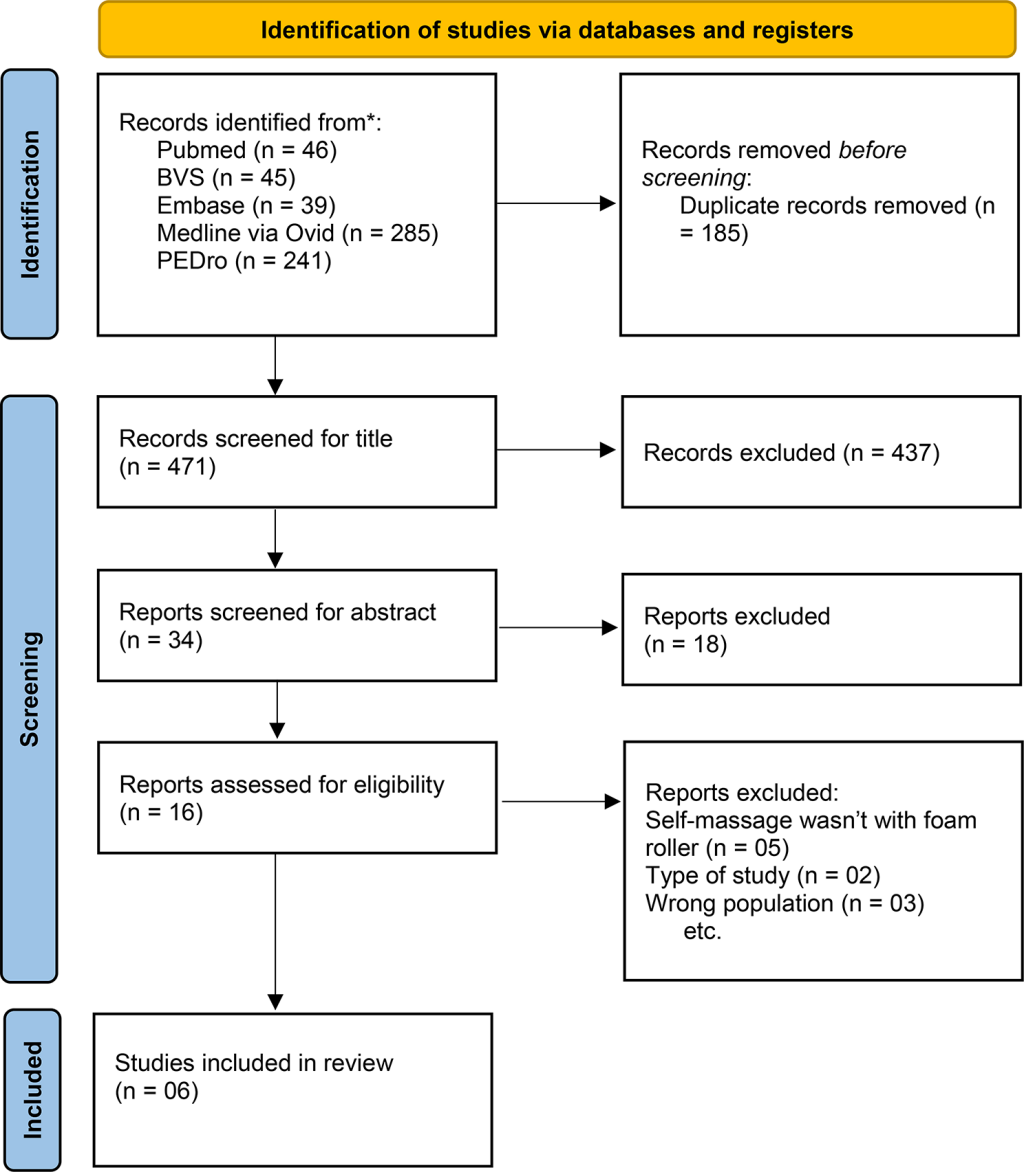
Selection of studies for inclusion in the systematic review

Using the previously defined search strategy, 656 manuscripts were obtained. However, after checking duplicates, titles, abstracts, complete reading, and implementation of eligibility criteria, only six manuscripts [19–25] were eligible for data analysis (Fig. 1).

### Description of studies

The studies included in the systematic review were published between 2018 and 2023. Altogether, the included manuscripts include the participation of 234 individuals (111 female, 121 male) with an average of 39 ± 7.23. With a minimum of 30 and a maximum of 50 individual study participants. With an age range between 17 and 74 (Table 1).

**Table 1:** General characteristics of the studies included

Study	Number of participants/ Gender	Age	Sample characteristics	Evaluation time	Pain Assessment	Therapy program	Frequency of treatment	Experimental Group	Control Group
Kumar et al. [19]	40 participants 0 males 40 females	Between 17–25 years, NR mean and standard deviation	Unilateral patellofemoral pain syndrome	4 weeks	VAS (10 cm)	FR + swiss ball vs. SLR	4 weeks Total of 56 sessions	10 reps daily of swiss ball exercises and 1 min of FR (FR applied twice a day)	SLR exercises for 10 reps daily
Ozsoy et al. [20]	42 participants 30 males 12 females	68.09 ± 2.77 years	Non-specific low back pain	6 weeks	VAS (10 cm),	Core stabilization exercises + hot pack + TENS vs. Core stabilization exercises + FR + hot pack + TENS	6 weeks Total of 18 sessions	Core exercises 1 to 3 sets, 8 to 15 reps and contractions from 5 to 10 s with 30s rests FR for 30s on each muscle area, 3 sets with 1 min rest Hot pack for 15 min TENS a 50-Hz with pulse < 150 µs 3 days per week	Core exercises 1 to 3 sets, 8 to 15 reps and contractions from 5 to 10 s with 30s rests hot pack for 15 min TENS a 50-Hz with pulse < 150 µs 3 days per week
Cabrera-Martos et al. [23]	40 participants 40 males 0 females	30.67 ± 5.23 years	Chronic neck pain	4 weeks	VAS (10 cm)	FR + roller balls + active upper limb neurodynamic exercises vs. booklet with information on neck pain	4 weeks Total of 12 sessions	50-to-60 min session with 15 min on each muscle area, 3 days per week	A booklet with information on neck pain
Ranbhor et al. [20]	50 participants 36 males 14 females	35.68 ± 12.25 years	Plantar fasciitis	1 day	VAS (10 cm)	FR vs. self-stretching	Total of 1 session	45 s per muscle area/5 reps with 15 s rest/1 day	45 s per muscle area/5 reps each rep more aggressive/1 day
Hameed et al. [22]	32 participants 11 males 21 females	25.75 ± 8.1 years	Plantar fasciitis	2 weeks	VAS (NR)	FR + ultrasound therapy vs. tennis ball + ultrasound therapy	2 weeks Total of 10 sessions	FR for 2 min Ultrassound 1.0 W/cm^2,^ on continuous mode and 3 MHz for 7 min 5 days per week	Tennis ball for 2 min Ultrassound 1.0 W/cm^2,^ on continuous mode and 3 MHz for 7 min 5 days per week
Yokochi et al. [24]	30 participants 4 males 26 females	74.3 ± 6.95 years	Total knee arthroplasty resulting from osteoarthritis	3 weeks	VAS (0-100 mm)	FR + regular physical therapy vs. regular physical therapy	3 weeks Total of 36 sessions	FR started 1 week after surgery with 60 s/3 reps on each muscle area/ twice a day/6 days per week Wheelchair practice, ROM exercises, strength training, walking exercise. 40 min each session/twice a day/6 days per week	Started 1 day after surgery/ Wheelchair practice, ROM exercises, strength training, walking exercise. 40 min each session/twice a day/6days per week

NR: Not reported, SLR: Straight leg raise technique, VAS: Visual analog scale, PPT: Pain Pressure Threshold, cm: centimeters, mm: millimeters, TENS: transcutaneous electrical stimulation, µs: microsecond, secs: seconds, W: Watts, Hz: Hertz, FR: Foam roller, reps: repetitions, ROM: Range of motion

Most manuscripts (5 of the 6) consisted of individuals with chronic musculoskeletal pain: Unilateral patellofemoral pain syndrome [19], Non-specific low back pain [21], Chronic neck pain [23], Plantar fasciitis [20, 22]. In only one of the manuscripts [24], the individuals had a condition characterized as acute pain established by total knee arthroplasty resulting from osteoarthritis.

### Methodological quality

Only two manuscripts [22, 24] did not have scores reported in the PEDro database. For this reason, two independent reviewers used the PEDro scale to score these manuscripts.

Using the PEDro scale, scores were assigned that varied between 4 and 8 points, with an average of 6 ± 1.29 points (Table 2). The PEDro scale criteria: random allocation, baseline comparability, adequate follow-up, point estimates, and variability were scored in all manuscripts, specifically about the criteria between-group comparisons and Intention-to-treat analysis. For the first, no score was assigned in only one of the manuscripts [23]. Regarding the second, only one manuscript received a score [23]. The criteria: Blind subjects, therapists, and Intention-to-treat analysis were not scored in any of the included manuscripts.

**Table 2:** Methodological evaluation of studies with technical specifications by PEDro scale

Study	Random allocation	Concealed allocation	Baseline comparability	Blind subjects	Blind therapists	Blind assessors	Adequate follow-up	Intention-to-treat analysis	Between-group comparisons	Point estimates and variability	Total score
Kumar et al. [19]	1	0	1	0	0	0	1	0	1	1	5
Ozsoy et al. [21]	1	0	1	0	0	1	1	0	1	1	6
Cabrera-Martos et al. [23]	1	1	1	0	0	1	1	1	1	1	8
Ranbhor et al. [20]	1	0	1	0	0	1	1	0	1	1	6
*Hameed et al. [22]	1	1	1	0	0	1	1	0	1	1	7
*Yokochi et al. [24]	1	0	1	0	0	0	1	0	0	1	4

0 = No, 1 = Yes, * Not reported on PEDro

### Interventions

The type of FR used was non-vibrational, being applied by a therapist in only one of the manuscripts [24]. With an application time ranging from at least 45 s [20] to 15 min [23], the non-vibrational FR was applied within a day [20] up to six weeks [21] (Table 3).

**Table 3:** Foam roller therapy characteristics

Study	Type of foam roller	Characteristics of foam roller	Time	Application mode	body region	Combined therapy
Kumar et al. [19]	Non-vibrational FR	1 feet long and 6 inches in diameter	1 min, twice a day/7 days per week/4 weeks	Self-application	Hip to knee (anteriorly, laterally, medially, posteriorly)	Swiss ball
Ozsoy et Al. [21]	Non-vibrational FR	Theraband®, The Hygenic Corporation, Akron, OH	30 s on each muscle with 1 min rest during 3 sets/3 days per week/6 weeks	After exercise/Therapist-application	Superficial back line (plantar fascia and short toe flexors, gastrocnemius/Achilles tendon, hamstrings and sacrolumbar fascia/erector spinae)	Core stabilization exercises + hot pack + TENS
Cabrera-Martos et al. [23]	Non-vibrational FR	Plastic cylinder and ball, with an outer covering of dense foam	15 min on each muscle area, 3 days per week/4 weeks	Before exercises/Self-application	Upper cervical spine, lower cervical spine, upper back and neck muscles	Active upper limb neurodynamic exercises
Ranbhor et al. [20]	Non-vibrational FR	NR	45 s per muscle area, 5 reps with 15 s rest/1 day	Self-application	Calf muscles and plantar fascia	-
Hameed et al. [22]	Non-vibrational FR	NR	2 min, 5 days per week/2 weeks	Self-application	Metatarsals to medial arch	Ultrasound therapy
Yokochi et al. [24]	Non-vibrational FR	TheraBand®, Akron, OH, USA	60 s, 3 reps on each muscle area, twice a day, 6 days per week/3 weeks	Before exercise/Therapist-application	Anterior, medial and lateral thigh	Regular physical therapy

NR: Not reported, secs: seconds, FR: Foam roller, reps: repetitions, ROM: Range of motion, TENS: transcutaneous electrical stimulation

Overall, non-vibrational FR was associated with other interventions in five manuscripts. Therapeutic exercises [19, 24], multimodal intervention protocol [21], manual therapy [23], therapeutic ultrasound [22]. Only one of the manuscripts used FR as a sole intervention [20]. Of these, only 3 described the therapeutic application window before exercises [23], and after [24] (Table 3).

### Outcomes measures and effect of interventions

At short-term follow-up, three studies [19, 23, 24] compared the use of FR associated with a therapeutic exercise program versus a therapeutic exercise program [19, 24], and versus therapeutic guidelines [23]. The following scales were used: VAS (10 cm) [19, 23], and VAS (0-100 mm) [24]. Kumar et al. [19] and Cabrera-Martos et al. [23] showed a statistically significant reduction in pain intensity when using FR associated with a therapeutic exercise program. Presenting, respectively, the values MD 2.4 (95% CI 1.94 to 2.86, and MD 2 (95% CI 0.64 to 3.36) (Table 4).

**Table 4:** Results and conclusions of studies regarding pain intensity

Study	Characterictics	Duration of treatment	Period of follow-up	Control group post-intervention (mean ± standard deviation)	Experimental group post-intervention (mean ± standard deviation)	Results	Conclusions
Kumar et al. [19]	*n* = 40 Unilateral patellofemoral pain syndrome. Non-vibrational FR. Combined with a swiss ball. VAS (10 cm)	4 weeks Total of 56 sessions	Short time follow-up	4.05 ± 0.759	1.65 ± 0.671	MD 2.4 (95% CI 1.94 to 2.86) in favor of the experimental group	Author: Foam combined with swiss ball reduced pain. Review: Foam combined with swiss ball reduced the intensity of pain.
Ozsoy et al. [21]	*n* = 42 NSLBP. Non-vibrational FR. Combined with CSE + hot pack + TENS. VAS (10 cm)	6 weeks Total of 18 sessions	Medium time follow-up	VAS at rest: 1.30 ± 1.13 VAS during activity: 3.37 ± 1.01	VAS at rest: 1.50 ± 1.30 VAS during activity: 3.73 ± 1.51	VAS at rest: MD -0.2 (95% CI -0.96 to 0.56) no statistically significant difference between groups VAS during activity: MD -0.36 (95% CI -1.16 to 0.44) no statistically significant difference between groups	Author: The current study suggests that myofascial release technique with a roller massager combined with core stabilization exercises can be a better choice in the treatment of NSLBP in elderly. Review: no statistically significant difference between groups for intensity of pain at rest and during activity.
Cabrera-Martos et al. [23]	*N* = 40 Chronic neck pain. Non-vibrational FR. Combined with active upper limb neurodynamic exercises. VAS (10 cm)	4 weeks Total of 12 sessions	Short time follow-up	6.00 ± 2.00	4.00 ± 2.25	MD 2 (95% CI 0.64 to 3.36) in favor of experimental group post-intervention	Author: A 4-week self-administered program for patients with chronic neck pain was effective in reducing the presence of active trigger point. Pain severity, average pain, and some aspects of functionality also improved significantly after the intervention. Review: Non-vibrational FR Combined with active upper limb neurodynamic exercises reduce intensity of pain.
Ranbhor et al. [20]	*n* = 50 Plantar fasciitis. Non-vibrational FR. VAS (10 Cm)	Total of 1 session	Immediately time	2.748 ± 1.68	2.496 ± 1.16	MD 0.252 (95% CI -0.57 to 1.07) no statistically significant difference between groups	Author: stretching and foam rolling techniques helped in reducing pain and increasing the ROM. However, the effectiveness of foam rolling was superior to stretching in terms of increase in the pain pressure threshold at gastrocnemius and soleus. Review: no statistically significant difference between groups.
Hameed et al. [22]	*n* = 32 Plantar fasciitis. Non-vibrational FR. Combined with ultrasound therapy. VAS (NR)	2 weeks Total of 10 sessions	Short time follow-up	3.81 ± 1.222	4.2 ± 1.294	MD -0.39 (95% CI -1.30 to 0.52) no statistically significant difference between groups	Author: Foam and tennis ball reduced pain, no statistically significant difference between groups. Review: no statistically significant difference between groups.
Yokochi et al. [24]	*n* = 30 Total knee arthroplasty resulting from osteoarthritis. Non-vibrational FR. Combined with Regular physical therapy. VAS (0-100 mm)	3 weeks Total of 36 sessions	Short time follow-up	VAS at rest: 5.3 ± 13.6 VAS during stretch: 17.7 ± 15.8	VAS at rest: 1.3 ± 3.0 VAS during stretch: 12.4 ± 19.7	VAS at rest: MD 4 (95% CI -3.37 to 11.37) no statistically significant difference between groups VAS during stretch: MD 5.3 (95% CI -8.06 to 18.66) no statistically significant difference between groups	Author: Compared with the control group, the FR intervention program significantly improved knee pain at stretching (knee flexion), but there was no synergistic effect on the other parameters. Review: no statistically significant difference between groups.

NR: Not reported, VAS: Visual analog scale, FR: Foam roller, ROM: Range of motion, MD: median; CI: confidence interval, CSE: core stabilization exercise, NSLBP: Non-specific low back pain, mm: millimeters, cm: centimeters, TENS: transcutaneous electrical stimulation

Also, at a short follow-up using the VAS, Hameed et al. [22] compared the use of FR associated with therapeutic ultrasound versus FR placebo with therapeutic ultrasound. However, no statistically significant differences between groups were reported (Table 4).

At medium-time follow-up, Ozsoy et al. [21] compared the use of FR associated with a multimodal protocol of therapeutic interventions versus a multimodal protocol of therapeutic interventions. Using the VAS at rest and during activity scale for this. However, no statistically significant differences between groups for pain intensity at rest and during activity were found (Table 4).

Ranbhor et al. [20] was the only study that compared FR without associations versus therapeutic allotment at immediate time follow-up using the VAS (10 cm). However, no statistically significant difference between groups was found (Table 4).

## Discussion

This review had the practical objective of summarizing the evidence on the effect of FR in individuals with chronic and acute musculoskeletal pain. Given the reviews previously carried out on the use of FR for indirect markers of muscle damage in healthy individuals [24], movement, muscle recovery, and performance [3, 13, 14, 26–29], range of motion for ankle dorsiflexion in healthy adults [30], Motion, muscle recovery, performance associated with exercise programs focused on stretching [31, 32] and use of vibrational FR on the range of motion and performance of normal individuals [33, 34]. This is the first systematic review that summarizes the results of studies using vibrational or non-vibrational FR on the outcome variable pain intensity in individuals with chronic and acute musculoskeletal pain.

Thus, of the six clinical trials included, four were characterized as short-term follow-ups [189 22, 23, 24], one medium-time follow-up [21] and immediate time follow-up [20]. Five used FR associated with other types of therapeutic exercise. Three randomized clinical trials estimated the effect of FR associated with a therapeutic exercise protocol [19, 23, 24]. Ozsoy et al. [21], used a multimodal therapeutic intervention protocol. Ranbhor et al. [20], used it associated with a stretching exercise protocol. Hameed et al. [22], associated with therapeutic ultrasound. Only Ranbhor et al. [20] used FR without associations with other interventions or therapeutic protocols. However, only two randomized clinical trials found a significant benefit in pain intensity of adding FR associated with a therapeutic exercise protocol in individuals with patellofemoral pain syndrome [19] and chronic neck pain [23].

Despite the benefits found in using FR associated with a therapeutic exercise protocol, there is a notable mix of the applicability of FR. Both used the VAS (10 cm) for evaluation, self-application, non-vibrational FR, and four weeks to carry out the interventions in individuals with two conditions related to chronic pain. The exposure intensities of the interventions were highly different, with ten daily repetitions [19] and 3 days per week [23]. This makes it unfeasible and challenging to define the best periodicity for the applicability of FR to reduce pain intensity. However, it demonstrates that better results from applying FR may be conditioned by therapeutic exercise protocols with a longer therapeutic window of exposure of at least four weeks.

The results highlighted in this review confirm the applicability trend of FR associated with therapeutic exercise protocols, such as stretching [31]. However, a recent systematic review and meta-analysis Konrad et al. [32] attested to significant heterogeneity in the applicability parameters of the FR in the studies analyzed, also found in this review. Furthermore, using FR and stretching protocols does not cause additional effects on the range of motion, only in athletes’ performance (e.g., strength, speed). Therefore, these results, added to the results presented in this systematic review on pain intensity, may be fundamental for structuring new clinical trials aiming to analyze the effects of FR associated with therapeutic exercise protocols based on stretching or muscle strengthening. The analysis of variables related to pain behavior, performance, and/or functional capacity in individuals with chronic musculoskeletal conditions should be taken as a basis. However, these new studies must attempt to standardize the time and intensity of FR applicability.

Far beyond the diversity of characteristics related to the applicability of the FR, it was possible to verify that, on average, randomized clinical trials had a methodological quality between fair and good on the PEDro scale [35]. However, in addition to the PEDro scale criteria, it should be noted that the manuscripts should have reported the prior registration of study protocols. Four of the six manuscripts [20, 21, 23, 24] described the sample size calculation. However, the previous description of these studies’ sample calculations must be completed. These methodological characteristics provide critical gaps regarding the transparency of the protocols used and even the existence of a relevant sampling power to reject or accept the hypotheses formulated by these studies.

When observing the results of the most recent systematic reviews on using FR, it is possible to attest to a mixed bag of results. The use of FR did not demonstrate any detrimental effect on improving flexibility. However, it appears to be effective for increasing the range of motion in a healthy adult population in the short term, up to 30 min [30]. And yet, even without promoting changes in markers related to indirect recovery from muscle damage (muscle pain, range of movement, muscle swelling, and maximum voluntary isometric contraction) in healthy individuals. It has excellent potential to increase jumping performance, agility, and strength and improve recovery Alonso-Calvete et al. [34] despite the absence of significant changes in performance when FR training is applied over one or several weeks [6, 36]. However, what stands out about these results is that they were based on the analysis of healthy individuals. Therefore, without health conditions that limit movement, flexibility, or functional performance. The results presented in this review, even if embryonic and preliminary, can be encouraging for the promotion of new randomized clinical trials aimed at using RF for acute and mainly chronic musculoskeletal conditions such as patellofemoral pain syndrome analyzed by Kumar et al. [19] and Chronic neck pain by Cabrera-Martos et al. [23].

Although limited and reinforces the need for more solid evidence for using FR concerning pain intensity, the results of this review provide space for future studies. Mainly, randomized clinical trials aimed at using FR to analyze pain-related variables in individuals with musculoskeletal conditions and with the appropriate size of participants. Using FR in the multimodal intervention context, therapeutic exercise protocols based on muscle strengthening or stretching are applied long-term, greater than or equal to 4 weeks. Seeking to structure the form of application between 1 and 15 min. With comparisons of the applicability of FR before or after therapeutic exercise protocols based on muscle strengthening or stretching. And with the confrontation of types of FR, non-vibrational versus vibrational. Finally, this review was conducted with searches in prominent research bases. However, not all databases available in the literature were used due to territorial access impossibilities. We recommend that future reviews structure a broader and more complete search using as many research bases as possible.

## Conclusion

Despite being promising and opening space for new studies, the results of this systematic review do not elucidate or reinforce the clinical use of FR in pain intensity in individuals with chronic and acute musculoskeletal pain.

## Data Availability

The datasets generated and/or analyzed during the current study are private due to our limited digital data stores for collective access. Still, they are available from the corresponding author on reasonable request.

## References

[1] Young JD, Spence AJ, Behm DG (2018). Roller massage decreases spinal excitability to the soleus. J Appl Physiol (1985).

[2] Pearcey GE, Bradbury-Squires DJ, Kawamoto JE, Drinkwater EJ, Behm DG, Button DC (2015). Foam rolling for delayed-onset muscle soreness and recovery of dynamic performance measures. J Athl Train.

[3] Wiewelhove T, Döweling A, Schneider C (2019). A Meta-analysis of the effects of Foam Rolling on Performance and Recovery. Front Physiol.

[4] Bradbury-Squires DJ, Noftall JC, Sullivan KM, Behm DG, Power KE, Button DC (2015). Roller-massager application to the quadriceps and knee-joint range of motion and neuromuscular efficiency during a lunge. J Athl Train.

[5] Shu D, Zhang C, Dai S, Wang S, Liu J, Ding J (2021). Acute effects of Foam Rolling on Hamstrings after Half-Marathon: a muscle functional magnetic resonance imaging study. Front Physiol.

[6] Pagaduan JC, Chang SY, Chang NJ (2022). Chronic effects of Foam Rolling on Flexibility and performance: a systematic review of Randomized controlled trials. Int J Environ Res Public Health.

[7] Pablos A, Ceca D, Jorda A (2020). Protective effects of Foam Rolling against inflammation and Notexin Induced Muscle Damage in rats. Int J Med Sci.

[8] Wilke J, Müller AL, Giesche F, Power G, Ahmedi H, Behm DG (2020). Acute effects of Foam Rolling on Range of Motion in healthy adults: a systematic review with Multilevel Meta-analysis. Sports Med.

[9] de Souza A, Sanchotene CG, Lopes CMDS (2019). Acute Effect of 2 self-myofascial release protocols on hip and ankle range of motion. J Sport Rehabil.

[10] Aboodarda SJ, Greene RM, Philpott DT, Jaswal RS, Millet GY, Behm DG (2018). The effect of rolling massage on the excitability of the corticospinal pathway. Appl Physiol Nutr Metab.

[11] Cavanaugh MT, Döweling A, Young JD (2017). An acute session of roller massage prolongs voluntary torque development and diminishes evoked pain. Eur J Appl Physiol.

[12] Page MJ, McKenzie JE, Bossuyt PM (2021). The PRISMA 2020 statement: an updated guideline for reporting systematic reviews. BMJ.

[13] Konrad A, Nakamura M, Behm DG (2022). The effects of Foam Rolling Training on Performance parameters: a systematic review and Meta-analysis including controlled and randomized controlled trials. Int J Environ Res Public Health.

[14] Konrad A, Nakamura M, Tilp M, Donti O, Behm DG (2022). Foam Rolling Training effects on Range of Motion: a systematic review and Meta-analysis. Sports Med.

[15] Watson JA, Ryan CG, Cooper L (2019). Pain Neuroscience Education for adults with Chronic Musculoskeletal Pain: a mixed-methods systematic review and Meta-analysis. J Pain.

[16] Maher CG, Sherrington C, Herbert RD, Moseley AM, Elkins M (2003). Reliability of the PEDro scale for rating quality of randomized controlled trials. Phys Ther.

[17] Macedo LG, Elkins MR, Maher CG, Moseley AM, Herbert RD, Sherrington C (2010). There was evidence of convergent and construct validity of Physiotherapy evidence database quality scale for physiotherapy trials. J Clin Epidemiol.

[18] Shiwa SR, Costa LO, Costa Lda C (2011). Reproducibility of the Portuguese version of the PEDro Scale. Cad Saude Publica.

[19] Kumar AS, Mohan Kumar G, Senthil Nathan CV, Rajalaxmi V, Ramachandran S, Veena Kirthika S, Sudhakar S, Padmanabhan K, Yuvarani G. Comparative analysis of Swiss ball and foam roller usage with slr technique for hip muscle strengthening of female athletes with patellofemoral pain syndrome. Biomed 2018; Apr-Jun;38(2):219–24.

[20] Ranbhor AR, Prabhakar AJ, Eapen C (2021). Immediate effect of foam roller on pain and ankle range of motion in patients with plantar fasciitis: a randomized controlled trial. Hong Kong Physiother J.

[21] Ozsoy G, Ilcin N, Ozsoy I (2019). The effects of myofascial release technique combined with Core Stabilization Exercise in Elderly with non-specific low back Pain: a randomized controlled, single-blind study. Clin Interv Aging.

[22] Hameed FS, Srivastava S. Effect of Self Myofascial Release using Foam Roller Versus Tennis ball in subjects with Plantar Fasciitis: a comparative study. Indian J Public Health Dev. 2020; 11(2).

[23] Cabrera-Martos I, Rodríguez-Torres J, López-López L, Prados-Román E, Granados-Santiago M, Valenza MC (2022). Effects of an active intervention based on myofascial release and neurodynamics in patients with chronic neck pain: a randomized controlled trial. Physiother Theory Pract.

[24] Yokochi M, Nakamura M, Iwata A (2023). A 1-Week Comprehensive Foam Rolling intervention program can improve knee Pain but not muscle function and range of motion in patients with total knee arthroplasty. Int J Environ Res Public Health.

[25] Medeiros F, Martins W, Behm D (2023). Acute effects of foam roller or stick massage on indirect markers from exercise-induced muscle damage in healthy individuals: a systematic review and meta-analysis. J Bodyw Mov Ther.

[26] Cheatham SW, Kolber MJ, Cain M, Lee M (2015). The effects of self-myofascial release using a foam rool or roller massager on joint range of motion, muscle recovery, and performance: a systematic review. Int J Sports Phys Ther.

[27] Beardsley C, Škarabot J (2015). Effects of self-myofascial release: a systematic review. J Bodyw Mov Ther.

[28] Hughes GA, Ramer LM (2019). Duration of myofascial rolling for optimal recovery, range of motion, and performance: a systematic review of the literature. Int J Sports Phys Ther.

[29] Skinner B, Moss R, Hammond L (2020). A systematic review and meta-analysis of the effects of foam rolling on range of motion, recovery and markers of athletic performance. J Bodyw Mov Ther.

[30] Grieve R, Byrne B, Clements C, Davies LJ, Durrant E, Kitchen O (2022). The effects of foam rolling on ankle dorsiflexion range of motion in healthy adults: a systematic literature review. J Bodyw Mov Ther.

[31] Konrad A, Nakamura M, Bernsteiner D, Tilp M (2021). The Accumulated effects of Foam Rolling combined with stretching on Range of Motion and physical performance: a systematic review and Meta-analysis. J Sports Sci Med.

[32] Konrad A, Tilp M, Nakamura M (2021). A comparison of the effects of Foam Rolling and stretching on physical performance. A systematic review and Meta-analysis. Front Physiol.

[33] Park SJ, Lee SI, Jeong HJ, Kim BG (2021). Effect of vibration foam rolling on the range of motion in healthy adults: a systematic review and meta-analysis. J Exerc Rehabil.

[34] Alonso-Calvete A, Lorenzo-Martínez M, Padrón-Cabo A (2022). Does Vibration Foam Roller Influence Performance and Recovery? A systematic review and Meta-analysis. Sports Med Open.

[35] Cashin AG, McAuley JH (2020). Clinimetrics: Physiotherapy evidence database (PEDro) scale. J Physiother.

[36] Hendricks S, Hill H, Hollander SD, Lombard W, Parker R (2020). Effects of foam rolling on performance and recovery: a systematic review of the literature to guide practitioners on the use of foam rolling. J Bodyw Mov Ther.

